# Concordance Between Watson for Oncology and a Multidisciplinary Clinical Decision-Making Team for Gastric Cancer and the Prognostic Implications: Retrospective Study

**DOI:** 10.2196/14122

**Published:** 2020-02-20

**Authors:** Yulong Tian, Xiaodong Liu, Zixuan Wang, Shougen Cao, Zimin Liu, Qinglian Ji, Zequn Li, Yuqi Sun, Xin Zhou, Daosheng Wang, Yanbing Zhou

**Affiliations:** 1 Department of Gastrointestinal Surgery The Affiliated Hospital of Qingdao University Qingdao University Qingdao China; 2 Department of Endocrinology Weifang People's Hospital Weifang China; 3 Department of Medical Oncology The Affiliated Hospital of Qingdao University Qingdao University Qingdao China; 4 Department of Imaging The Affiliated Hospital of Qingdao University Qingdao University Qingdao China

**Keywords:** Watson for Oncology, artificial intelligence, gastric cancer, concordance, multidisciplinary team

## Abstract

**Background:**

With the increasing number of cancer treatments, the emergence of multidisciplinary teams (MDTs) provides patients with personalized treatment options. In recent years, artificial intelligence (AI) has developed rapidly in the medical field. There has been a gradual tendency to replace traditional diagnosis and treatment with AI. IBM Watson for Oncology (WFO) has been proven to be useful for decision-making in breast cancer and lung cancer, but to date, research on gastric cancer is limited.

**Objective:**

This study compared the concordance of WFO with MDT and investigated the impact on patient prognosis.

**Methods:**

This study retrospectively analyzed eligible patients (N=235) with gastric cancer who were evaluated by an MDT, received corresponding recommended treatment, and underwent follow-up. Thereafter, physicians inputted the information of all patients into WFO manually, and the results were compared with the treatment programs recommended by the MDT. If the MDT treatment program was classified as “recommended” or “considered” by WFO, we considered the results concordant. All patients were divided into a concordant group and a nonconcordant group according to whether the WFO and MDT treatment programs were concordant. The prognoses of the two groups were analyzed.

**Results:**

The overall concordance of WFO and the MDT was 54.5% (128/235) in this study. The subgroup analysis found that concordance was less likely in patients with human epidermal growth factor receptor 2 (HER2)-positive tumors than in patients with HER2-negative tumors (*P*=.02). Age, Eastern Cooperative Oncology Group performance status, differentiation type, and clinical stage were not found to affect concordance. Among all patients, the survival time was significantly better in concordant patients than in nonconcordant patients (*P*<.001). Multivariate analysis revealed that concordance was an independent prognostic factor of overall survival in patients with gastric cancer (hazard ratio 0.312 [95% CI 0.187-0.521]).

**Conclusions:**

The treatment recommendations made by WFO and the MDT were mostly concordant in gastric cancer patients. If the WFO options are updated to include local treatment programs, the concordance will greatly improve. The HER2 status of patients with gastric cancer had a strong effect on the likelihood of concordance. Generally, survival was better in concordant patients than in nonconcordant patients.

## Introduction

Gastric cancer is a common malignant tumor worldwide. Its prognosis is relatively poor, and it is a serious threat to human health. According to the Global Cancer Statistics 2018, there were approximately 1.03 million new gastric cancer cases and approximately 728,685 deaths, and gastric cancer ranked fifth in incidence and third in mortality among malignant tumors [1]. China has a large number of patients with gastric cancer, with annual new cases accounting for more than 40% of the cases worldwide, and gastric cancer is the most commonly diagnosed gastrointestinal cancer [2]. Therefore, enhancing the diagnosis and treatment of gastric cancer and improving the survival of patients are urgent goals for experts and scholars in China.

With the development of modern medicine, the methods of cancer treatment are becoming increasingly abundant. New technologies, ideas, drugs, and programs are emerging. It is difficult to provide a reasonable and scientific treatment program for patients by relying on only one specific modality. It is necessary to change the individualized treatment model from a “single soldier combat” model to a “multidisciplinary cooperation” model. Multidisciplinary teams (MDTs) have become an inevitable trend in the development of oncology [3]. The National Comprehensive Cancer Network Panel believes in an infrastructure that encourages multidisciplinary treatment decision-making by members of all disciplines taking care of this group of patients. Through multidisciplinary team consultation, gastric cancer patients can receive the best comprehensive treatment.

The development of artificial intelligence (AI) technology is speeding up, and its application in the medical domain is increasing. Scientists and clinicians are working together to leverage machine learning and deep learning in drug discovery, imaging, pathology, genetic testing, and clinical decision support to improve productivity and accuracy with reduced cost. By 2025, it is estimated that up to US $54 billion in health-care costs will be saved globally per year owing to the impact of AI [4]. Currently, as one of the most representative AI supportive tools for cancer care, IBM Watson for Oncology (WFO) can help oncologists deal with explosively increasing evidence and provide a multidisciplinary treatment plan having high conformity and concordance with high-quality evidence according to patient information, which can play an essential role in eliminating the inequity of cancer care. Many clinical studies regarding precision medicine have promoted progress in the treatment of malignant tumors, such as gastric cancer, and have shortened the update cycle of guidelines. However, as knowledge is updated, the pressure on clinicians is increasing. One of the leading AI tools is WFO, which can deeply learn and understand the enormous body of literature available to the scientific community. AI can help make connections among all the data needed to answer a complex medical question in a short time. Moreover, AI, as a helpful assistant for oncologists, can build confidence among physicians and patients, improve the efficiency of physicians’ clinical decision-making, and promote the further development of evidence-based medicine and precision medicine [5]. There is a common need to improve decision-making time and the future of medicine.

There have been related reports on breast cancer [6-8], lung cancer [7-9], colorectal cancer [10], and other cancers, which have demonstrated high concordance between WFO and MDTs. However, research on gastric cancer has been limited so far. Therefore, our team conducted a retrospective study to evaluate the concordance between WFO and an MDT for patients with gastric cancer in order to explore the factors affecting concordance and the reasons for nonconcordance. Moreover, we compared patient prognosis between those with and those without this concordance.

## Methods

### Study Design and Patient Population

**Figure 1:**
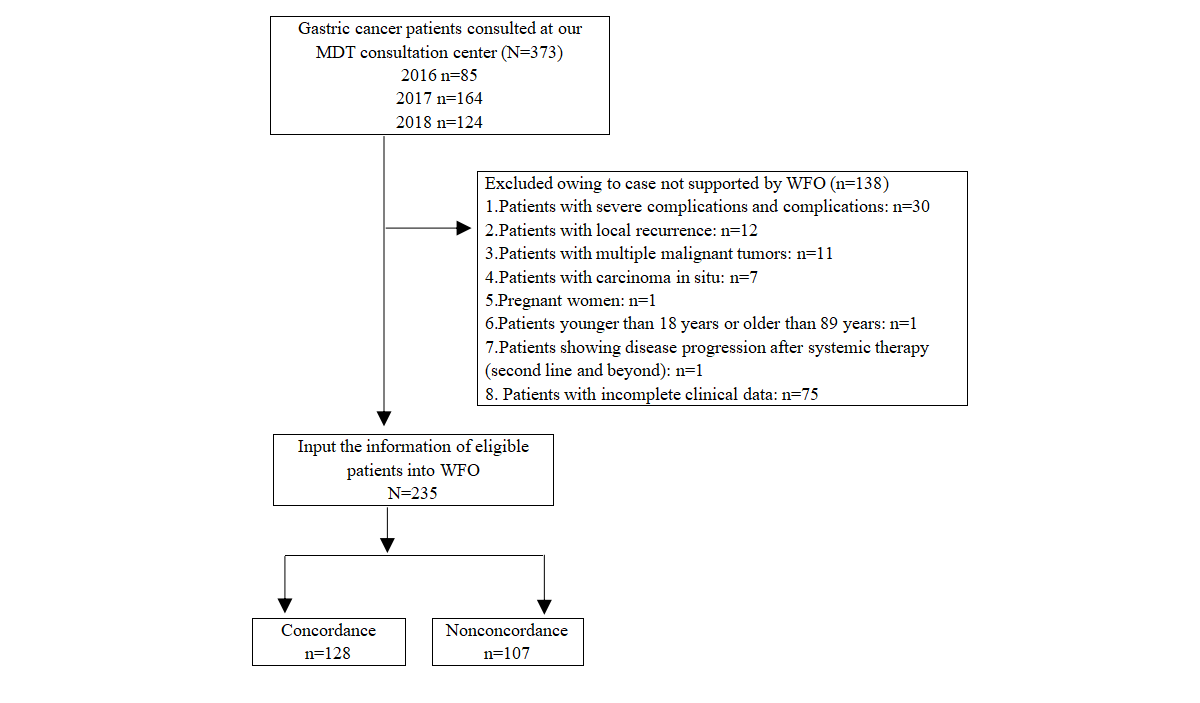
Flow diagram of the patient selection process. MDT: multidisciplinary team; WFO: Watson for Oncology.

This study selected patients with gastric cancer who were evaluated by the MDT board from January 2016 to June 2018 at the Affiliated Hospital of Qingdao University. The exclusion criteria were as follows: (1) incomplete clinical data; (2) carcinoma in situ; (3) pregnancy; (4) multiple concurrent primary cancers; (5) severe complications; (6) local recurrence; (7) age younger than 18 years or older than 89 years; and (8) participation in any clinical trial. A total of 373 patients were identified. Initially, 63 patients beyond the coverage scope of WFO were excluded, and thereafter, 75 patients with incomplete clinical data were excluded. A total of 235 patients were finally included in this study (Figure 1).

### Watson for Oncology

Patient information and specific treatment program information were collected from the hospital’s electronic case system, and two senior physicians, who were blinded to the actual treatment, manually entered the patient information into WFO (version 18.3, IBM Watson Health, Cambridge, Massachusetts) and recorded the WFO recommendations. Treatment recommendations from WFO were divided into the following three categories: recommended, for consideration, and not recommended. During the data analysis process, we found some actual treatment options that were not available in WFO, which were defined as “physician’s decision.” Our team compared the treatment recommendations given by WFO and the MDT. If an MDT treatment plan was classified by WFO as “recommended” or “for consideration,” it was considered concordant; otherwise, it was considered nonconcordant. The study protocol was approved by the Ethics Committee of the Affiliated Hospital of Qingdao University (QYFYKYLL 2018-34).

### Data Analysis and Statistics

We used SPSS 23.0 (IBM Corp, Armonk, New York) to describe the data and perform statistical analyses. To simultaneously control the determinants of concordance, a logistic regression model was estimated, and odds ratios and 95% confidence intervals were reported. The probability of overall survival was estimated by using the Kaplan-Meier method. The multivariate analysis used the Cox proportional hazard model. A *P* value <.05 was considered statistically significant.

## Results

### Concordance and Characteristics of the Patients With Gastric Cancer

When the treatment regimen of the MDT was compared with WFO decision-making, the results were as follows: recommended, 43.0% (101/235); for consideration, 11.5% (27/235); not recommended, 6.8% (16/235); and physician’s decision, 38.7% (91/235) (Table 1). Subgroup analyses of treatment concordance according to human epidermal growth factor receptor 2 (HER2) status and clinical stage were also carried out. The concordance rate was 56.1% (119/212) in HER2-negative patients and was 39% (9/23) in HER2-positive patients. The concordance differences observed according to clinical stage were as follows: stage I, 77% (10/13); stage II, 74% (17/23); stage III, 52.5% (64/122); and stage IV, 48% (37/77).

On comparing the treatment regimens, 107 patients were included in the nonconcordant group and 128 were included in the concordant group. There were no significant differences in clinical data between the two groups (Table 2).

**Table 1:** Treatment concordance between Watson for Oncology and the multidisciplinary team (N=235).

Concordant cases, n (%)			Nonconcordant cases, n (%)		
Recommended	For consideration	Total	Not recommended	Physician’s choice	Total
101 (43.0)	27 (11.5)	128 (54.5)	16 (6.8)	91 (38.7)	107 (45.5)

**Table 2:** Characteristics of the 235 study patients at baseline.

Characteristic		Total (N=235), n (%)	Concordance (n=128), n (%)	Nonconcordance (n=107), n (%)	χ² (*df*)	*P* value
**Age** **(years)**					2.1 (1)	.15
	<70	167 (71.1)	86 (67.2)	81 (75.7)		
	≥70	68 (28.9)	42 (32.8)	26 (24.3)		
**Gender**					0.2 (1)	.70
	Male	159 (67.7)	88 (68.8)	71 (66.4)		
	Female	76 (32.3)	40 (31.3)	36 (33.6)		
**BMI^a^**					2.3 (2)	.31
	<18.5	29 (12.3)	12 (9.4)	17 (15.9)		
	18.5-23.9	131 (55.7)	73 (57.0)	58 (54.2)		
	≥24	75 (31.9)	43 (33.6)	32 (29.9)		
**ECOG^b^** **PS^c^**					2.5 (2)	.29
	1	181 (77.0)	95 (74.2)	86 (80.4)		
	2	39 (16.6)	22 (17.2)	17 (15.9)		
	3	15 (6.4)	11 (8.6)	4 (3.7)		
**NRS^d^** **2002 PS^c^**					0.0 (1)	.98
	<3	92 (39.1)	50 (39.1)	42 (39.3)		
	≥3	143 (60.9)	78 (60.9)	65 (60.7)		
**Comorbidity**						
	Hypertension	55 (23.4)	28 (21.9)	27 (25.2)	0.4 (1)	.54
	Diabetes	25 (10.6)	12 (9.4)	13 (12.1)	0.5 (1)	.49
	Coronary heart disease	46 (19.6)	21 (16.4)	25 (23.4)	1.8 (1)	.18
	Abdominal surgery history	21 (8.9)	11 (8.6)	10 (9.3)	0.0 (1)	.84
**Tumor size (cm)**					1.6 (1)	.21
	<5	148 (63.0)	76 (59.4)	72 (67.3)		
	≥5	87 (37.0)	52 (40.6)	35 (32.7)		
**Lauren classification**					5.1 (2)	.08
	Intestinal type	85 (36.2)	53 (41.4)	32 (29.9)		
	Mixed type	84 (35.7)	46 (35.9)	38 (35.5)		
	Diffuse type	66 (28.1)	29 (22.7)	37 (34.6)		
**Helicobacter pylori**					0.9 (1)	.34
	Negative	144 (61.3)	82 (64.1)	62 (57.9)		
	Positive	91 (38.7)	46 (35.9)	45 (42.1)		
**Histologic type**					2.9 (1)	.09
	Well/moderate	44 (18.7)	29 (22.7)	15 (14.0)		
	Poor	191 (81.3)	99 (77.3)	92 (86.0)		
**HER2^e^** **status**					2.4 (1)	.12
	Negative	212 (90.2)	119 (93.0)	93 (86.9)		
	Positive	23 (9.8)	9 (7.0)	14 (13.1)		
**Tumor location**					1.9 (2)	.39
	Upper	69 (29.4)	35 (27.3)	34 (31.8)		
	Middle	47 (20.0)	23 (18.0)	24 (22.4)		
	Lower	119 (50.6)	70 (54.7)	49 (45.8)		
**T-stage**					6.3 (3)	.10
	T1	7 (3.0)	5 (3.9)	2 (1.9)		
	T2	16 (6.8)	13 (10.2)	3 (2.8)		
	T3	45 (19.1)	25 (19.5)	20 (18.7)		
	T4	167 (71.1)	85 (66.4)	82 (76.6)		
**N-stage**					6.6 (3)	.08
	N0	16 (6.8)	11 (8.6)	5 (4.7)		
	N1	44 (18.7)	29 (22.7)	15 (14.0)		
	N2	71 (30.2)	40 (31.3)	31 (29.0)		
	N3	104 (44.3)	48 (37.5)	56 (52.3)		
**M-stage**					1.9 (1)	.17
	M0	158 (67.2)	91 (71.1)	67 (62.6)		
	M1	77 (32.8)	37 (28.9)	40 (37.4)		
**cStage^f^**					7.6 (3)	.05
	I	13 (5.5)	10 (7.8)	3 (2.8)		
	II	23 (9.8)	17 (13.3)	6 (5.6)		
	III	122 (51.9)	64 (50.0)	58 (54.2)		
	IV	77 (32.8)	37 (28.9)	40 (37.4)		
**Previous therapies**					0.6 (1)	.44
	Yes	86 (36.6)	44 (34.4)	42 (39.3)		
	No	149 (63.4)	84 (65.6)	65 (60.7)		

^a^BMI: body mass index.

^b^ECOG: Eastern Cooperative Oncology Group.

^c^PS: performance status.

^d^NRS: nutrition risk screening.

^e^HER2: human epidermal growth factor receptor 2.

^f^cStage: clinical stage; TNM-8, the Union for International Cancer Control 8th edition and American Joint Committee on Cancer 8th edition.

### Nonconcordant Patients

In this study, nonconcordant patients accounted for 45.5% (107/235) of the study population. Among the nonconcordant patients, 74 patients received chemotherapy regimens that were not recommended by WFO (such as S-1 plus oxaliplatin [SOX]), 11 patients with stage IV cancer underwent surgical resection after systemic treatment (although WFO had recommended radiotherapy or systemic therapy until disease progression), and 11 patients were treated with chemotherapy only (although WFO had recommended chemotherapy combined with radiotherapy). In addition, 6 patients were treated with systemic therapy and oral apatinib, which is a small molecule antiangiogenic targeted drug, 3 patients underwent endoscopic therapy (although WFO recommended surgery), and 2 patients underwent hyperthermic intraperitoneal chemotherapy. Of the 74 patients who received nonconcordant chemotherapy regimens, 55 were treated with the SOX regimen, but WFO did not indicate this regimen, and 19 were treated with other chemotherapy regimens.

### Factors Influencing Concordance

Table 3 shows the results from the logistic regression analysis of concordance as a function of patient age, Eastern Cooperative Oncology Group performance status, differentiation type, HER2 status, clinical stage, and previous therapies. Only HER2 status (*P*=.02) had a significant effect on concordance.

**Table 3:** Logistic regression model of concordance between Watson for Oncology and the multidisciplinary team.

Variable		B	SE	Wald	OR (95% CI)	*P* value
**Age (years)**						
	<70 (reference)	—^a^	—	—	1.000	
	≥70	0.210	0.403	0.271	1.233 (0.560-2.715)	.60
**ECOG^b^** **PS^c^**						
	1 (reference)	—	—	0.668	1.000	.72
	2	−0.569	0.718	0.627	0.566 (0.139-2.314)	.43
	3	−0.534	0.704	0.574	0.586 (0.148-2.331)	.45
**Differentiation type**						
	Well/moderate (reference)	—	—	—	1.000	
	Poor	−0.407	0.370	1.211	0.666 (0.322-1.374)	.27
**HER2^d^** **status**						
	Negative (reference)	—	—	—	1.000	
	Positive	−1.028	0.440	5.461	0.358 (0.151-0.847)	.02
**cStage^e^**						
	Ⅰ (reference)	—	—	4.714	1.000	.19
	II	1.217	0.724	2.831	3.379 (0.818-13.951)	.90
	III	0.848	0.559	2.303	2.335 (0.781-6.978)	.13
	IV	0.103	0.300	0.117	1.108 (0.615-1.995)	.77
**Previous** **therapies**						
	Yes (reference)	—	—	—	1.000	
	No	−0.112	0.295	0.144	0.894 (0.501-1.594)	.70

^a^Not applicable.

^b^ECOG: Eastern Cooperative Oncology Group.

^c^PS: performance status.

^d^HER2: human epidermal growth factor receptor 2.

^e^cStage: clinical stage; TNM-8, the Union for International Cancer Control 8th edition and American Joint Committee on Cancer 8th edition.

### Prognostic Analysis

**Figure 2:**
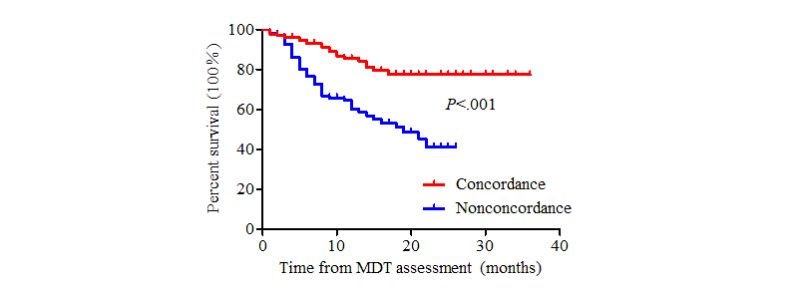
Overall survival in all patients. MDT: multidisciplinary team.

**Figure 3:**
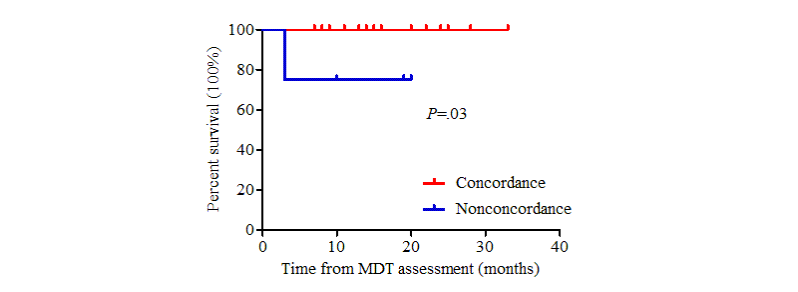
Overall survival in stage II patients. MDT: multidisciplinary team.

**Figure 4:**
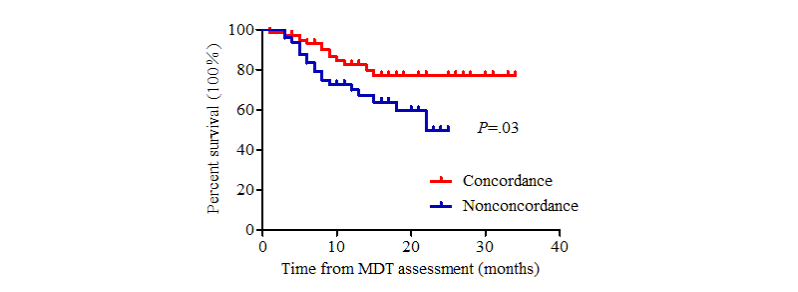
Overall survival in stage III patients. MDT: multidisciplinary team.

**Figure 5:**
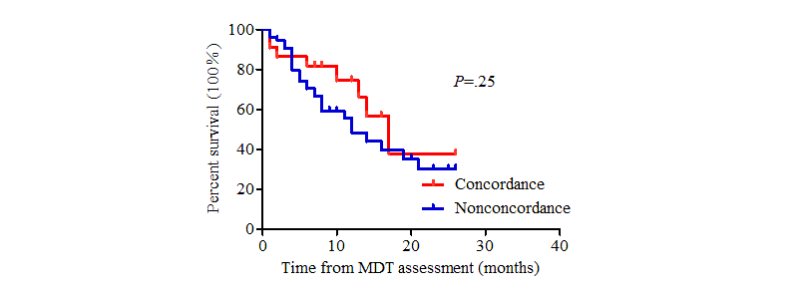
Overall survival in stage IV patients. MDT: multidisciplinary team.

The patients in this study were followed until January 31, 2019. In the concordant group, 49 patients received surgical treatment directly, 42 patients received neoadjuvant therapy before surgery, 36 patients received systematic treatment until the disease progressed, and 1 patient received symptomatic support treatment. The actual treatment regimens received in the nonconcordant group are presented above. Seventy patients died during follow-up. The average survival time was 16.4 months for nonconcordant patients and 30.0 months for concordant patients (log-rank test, χ^2^=22.6_1_, *P*<.001) (Figure 2). A stratified analysis was carried out according to disease stage. There was a significant difference between the two groups among patients with clinical stage II and III diseases (*P*=.03, Figure 3 and *P*=.03, Figure 4, respectively). By contrast, there was no significant difference in the survival curve between the two groups among patients with clinical stage IV disease (*P*=.25, Figure 5). Univariate prognostic analysis revealed that consistency and clinical stage were associated with overall survival in the patients with gastric cancer. We further performed a multivariate analysis and found that the same factors remained significant (Table 4).

**Table 4:** Multivariate analysis of patients with gastric cancer.

Variable		Univariate survival analysis				Multivariate analysis^a^			
		Hazard ratio (95% CI)		*P* value		Hazard ratio (95% CI)		*P* value	
Concordance (no/yes)		0.312 (0.187-0.521)		<.001		0.374 (0.220-0.634)		<.001	
Age (<70/≥70 years)		1.265 (0.771-2.075)		.35		—^b^		—	
Gender (male/female)		1.191 (0.730-1.943)		.48		—		—	
**ECOG^c^** **PS^d^**									
	1		0.719 (0.286-1.805)		.48		—		—
	2		1.092 (0.400-2.983)		.86		—		—
	3 (reference)		—		.31		—		—
NRS^e^ 2002 PS (<3/≥3)		1.231 (0.752-2.018)		.41		—		—	
Differentiation type (well, moderate/poor)		1.769 (0.878-3.563)		.11		1.166 (0.571-2.380)		.67	
HER2^f^ status (negative/positive)		1.681 (0.903-3.131)		.10		0.986 (0.517-1.881)		.97	
**cStage^g^**									
	I		0.000 (0.000-5.030)		.97		0.000 (0.001-9.960)		.97
	II		0.066 (0.009-0.481)		.01		0.087 (0.012-0.638)		.02
	III		0.400 (0.248-0.646)		<.001		0.417 (0.256-0.678)		<.001
	Ⅳ (reference)		—		<.001		—		.001

^a^Multivariate model included concordance, differentiation type, HER2 status, and clinical stage. Enter model selection was performed.

^b^Not applicable.

^c^ECOG: Eastern Cooperative Oncology Group.

^d^PS: performance status.

^e^NRS: nutrition risk screening.

^f^HER2: human epidermal growth factor receptor 2.

^g^cStage: clinical stage; TNM-8, the Union for International Cancer Control 8th edition and American Joint Committee on Cancer 8th edition.

## Discussion

### Principal Findings

Globally, to the best of our knowledge, this is the first article exploring both concordance and survival impact using WFO in patients with gastric cancer.

This study showed that the overall concordance of WFO and the MDT was 54.5%. Although the concordance was lower than that in published studies on breast cancer [6], lung cancer [8], and advanced gastric cancer from South Korea [11], our finding is similar to the concordance of 49% in gastric cancer identified in a gastrointestinal cancer study reported at the 2017 American Society of Clinical Oncology (ASCO) Annual Meeting [10]. To determine the impact of patient characteristics and treatment status on concordance, we performed logistic regression analysis, and the results showed that only HER2 status affected concordance. The concordance of HER2-positive patients was lower than that of HER2-negative patients. In addition, we found that concordance decreased as the patient stage changed from early to advanced; this observation requires a large sample size for further validation.

As there was a large proportion of patients receiving treatment that was not recommended by WFO, we looked further into the reasons for nonconcordance. First, in terms of fluoropyrimidine drugs, the standard program in the United States involves 5-fluorouracil or capecitabine. Owing to differences in patient characteristics and genomic background, Chinese clinical practice regarding gastric cancer has adopted more criteria from the Japanese guidelines, which have shown obvious benefits for patients [12]. China has adopted chemotherapy regimens involving S-1 capsules, such as SOX, and previous studies have found that the SOX regimen is similarly safe and effective for gastric cancer [13]. There were 55 patients treated with the SOX regimen, although WFO was not able to recommend this regimen. If WFO could recommend SOX as a reasonable alternative to capecitabine plus oxaliplatin, the overall concordance of WFO and the MDT would have increased from 54.5% (128/235) to 77.9% (183/235). Second, the application of targeted drugs and immune therapy is limited in China because of patients’ affordability, China’s medical reimbursement policy, and lack of approval by the China Food and Drug Administration. Third, for patients with locally advanced inoperable diseases, radiotherapy and chemotherapy are routinely used in the United States. However, owing to domestic equipment and technical limitations, as well as additional adverse effects and economic expenditure, the acceptance of domestic radiotherapy in China is generally low [14]. We are accustomed to prescribing chemotherapy alone to locally advanced patients. For advanced patients with distant metastases, WFO recommends systemic treatment until disease progression or symptomatic supportive care. However, we treat some patients with surgery after reaching partial or total remission (partial response or complete response), thus improving the prognosis. It has been reported that patients with unresectable gastric cancer who initially exhibit one noncurative factor may obtain a survival benefit from chemotherapy and subsequent curative surgery [15]. Fourth, in recent years, China’s first independently developed targeted drug apatinib has been proven to be effective as a third-line treatment for metastatic gastric cancer [16]. At the same time, we used hyperthermic intraperitoneal chemotherapy for some advanced patients [17], which is not available in the WFO system. The treatment recommendations offered by WFO are based more on the National Comprehensive Cancer Network guidelines and the treatment experiences of the Memorial Sloan Kettering Cancer Center. We can see that there are still differences in the treatment of gastric cancer between the United States and China. Local guidelines should be incorporated into WFO for better application in China.

In this study, we innovatively analyzed the relationship between concordance and survival. Our study found that survival was much better in concordant patients than in nonconcordant patients. Previous ASCO meetings reported that the survival of patients with stage I and III diseases in the concordant group was much better than the survival of patients with stage I and III diseases in the nonconcordant group [18]. In this study, there was no significant difference in the prognosis of patients with stage II disease between the concordant and nonconcordant groups, but the sample size was small. This observation needs to be further validated in larger samples. We found that the prognosis of the concordant group was much better than that of the nonconcordant group. At the same time, the treatment recommendations provided by WFO further confirmed the safety and effectiveness of incorporating AI. Patients with clinical stage III and IV diseases had complex conditions, and multidisciplinary comprehensive treatment was required. These patients often need the MDT the most. WFO provides the greatest support to the MDT, because it involves comprehensive knowledge that is based on evidence and weighs the opinions of multiple disciplines. WFO can help patients achieve a good prognosis.

This study has some limitations and shortcomings. First, we performed a retrospective analysis, the baseline differences between the groups and some subgroups could not be eliminated, and the sample size was small. All these factors may have caused bias regarding the results. Second, the treatment consensus may change over time to nonconcordance; however, owing to the heavy workload of oncologists and the large sample size needed, we have not yet organized a second blind trial. However, a previous study involving breast cancer [4] showed that concordance increased from 77% to 93% after a second blind trial of nonconcordant patients. Therefore, we believe that with the further study of updated guidelines and the accumulation of clinical experience, concordance will be higher if cases of gastric cancer are re-evaluated.

Although WFO has certain limitations in the treatment of gastric cancer, its advantages and development prospects cannot be ignored. First, oncologists face heavy clinical workload, limiting the time available for learning [19]. Therefore, facing the challenge of the rapid expansion of professional knowledge, oncologists urgently need a tool that can effectively study related fields and cutting-edge knowledge. WFO has the characteristic of the use of intensive learning with massive data, and it may help physicians improve their learning efficiency and the accuracy of their clinical decisions. Second, the modern medical model emphasizes democracy (ie, participants include physicians, patients’ families, and even society). However, the physician or patient may not choose the most appropriate standardized program owing to preference [20]. WFO has the characteristic of objective neutrality, and it provides a detailed list of the treatment programs according to evidence, which can ensure accuracy of decision-making. However, WFO lacks individualized considerations for patients and human care. Therefore, when physicians, patients, and WFO work together and maintain close coordination, they can make up for their respective shortcomings and achieve excellent and optimal care. Third, the imbalance of domestic medical resource allocation makes it difficult for patients at the grassroot level to obtain standardized treatment [21]. The emergence of WFO has enabled patients in primary hospitals to obtain the same standardized and personalized treatment plans as those in first-tier cities. Therefore, the continuous improvement and popularization of AI aids will help improve overall medical efficiency and quality and promote the development of evidence-based medicine and standardized treatment.

### Conclusions

The treatment programs in patients with gastric cancer were mostly concordant between WFO and the MDT. If WFO options are updated to include local treatment programs, the concordance will greatly improve. The HER2 receptor status had a strong effect on concordance. Prognosis was better among patients in the concordant group than among patients in the nonconcordant group. At present, WFO cannot completely replace clinicians, but it can be used as a tool to assist physicians.

## Abbreviations

AI: artificial intelligence
ASCO: American Society of Clinical Oncology
HER2: human epidermal growth factor receptor 2
MDT: multidisciplinary team
SOX: S-1 plus oxaliplatin
WFO: Watson for Oncology
